# Parental precarious employment and the mental health of adolescents: a Swedish registry study

**DOI:** 10.5271/sjweh.4210

**Published:** 2025-03-01

**Authors:** Amanda E Aronsson, Emelie Thern, Nuria Matilla-Santander, Signild Kvart, Julio C Hernando-Rodriguez, Kathryn Badarin, Mireia Julià, Samira Alfayumi-Zeadna, Virginia Gunn, Bertina Kreshpaj, Carles Muntaner, Theo Bodin, Lluís Mangot-Sala

**Affiliations:** 1 Centre for Global Health Inequalities Research (CHAIN), Department of Sociology and Political Science, Norwegian University of Science and Technology (NTNU), Trondheim, Norway.; 2 Unit of Occupational Medicine, Institute of Environmental Medicine, Karolinska Institutet, Stockholm, Sweden.; 3 Social Determinants and Health Education Research Group (SDHEd), Hospital del Mar Research Institute, Barcelona, Spain.; 4 Hospital del Mar Nursing School (ESIHMar), Universitat Pompeu Fabra-affiliated, Barcelona, Spain.; 5 Research Group on Health Inequalities, Environment, and Employment Conditions Network (GREDS), Universitat Pompeu Fabra, Barcelona, Spain.; 6 MAP Centre for Urban Health Solutions, Li Ka Shing Knowledge Institute, Unity Health Toronto, Toronto, Ontario, Canada; 7 School of Nursing, Cape Breton University, Sydney, Nova Scotia, Canada.; 8 Department of Public Health, University of Copenhagen, Copenhagen, Denmark.; 9 Bloomberg Faculty of Nursing, Division of Social and Behavioural Sciences, University of Toronto, Toronto, Ontario, Canada.; 10 JHU-UPF Public Policy Center (JHU-UPF PPC), Universitat Pompeu Fabra (UPF) - UPF Barcelona School of Management (UPF-BSM), Barcelona, Spain.

**Keywords:** adolescent health, health inequality, intergenerational transmission, longitudinal study, mental health, precarious job, precarity, register-based

## Abstract

**Objective:**

This study investigates the association between parental precarious employment (PE) and the mental health of their adolescent children, with a particular focus on how the association differs based on whether the mother or father is in PE.

**Methods:**

This register-based study used the Swedish Work, Illness, and Labor-market Participation (SWIP) cohort. A sample of 117 437 children aged 16 years at baseline (2005) were followed up until 2009 (the year they turned 20). A multidimensional construct of PE (SWE-ROPE 2.0) was used to classify parental employment as either precarious, substandard or standard. The outcome, adolescents’ mental disorders, was measured as a diagnosis of a mental disorder using ICD-10 codes or by prescribed psychotropic drugs using ATC codes. Crude and adjusted Cox regression models produced hazard ratios (HR) with 95% confidence intervals (CI) to estimate the association between parental PE and adolescents’ mental health.

**Results:**

Adolescents with parents in PE exhibited a higher risk of developing mental disorders. The association was more pronounced for paternal PE (HR 1.22, 95% CI 1.10–1.35) compared to maternal PE (HR 1.11, 95% CI 1.00–1.21). These associations largely persisted after adjusting for important confounders, including parental mental health.

**Conclusion:**

This study addresses a significant gap in the literature on parental PE and adolescents’ mental health. As PE is growing more common across countries, this study provides relevant insights into the intergenerational role that parental low-quality employment may have in terms of mental health within families.

Precarious employment (PE) is growing increasingly common in many countries and can have several health damaging effects (1) resulting, for instance, from increased stress, financial hardship and hazardous working conditions. PE is associated with several adverse health effects and health risks for the worker (2–4), including mental health issues (5–7), suicide attempts and substance use disorders (8). PE specifically refers to the (poor) quality of employment, it is a multidimensional construct measured by the elements of income inadequacy, employment insecurity and a lack of rights and protections (9). While it is known that PE can have negative effects on worker’s health, an increasing body of evidence suggests that PE and related factors, such as hazardous and poor psychosocial working conditions, can also harm the health and well-being of family members (1, 10). This implies that there may be an intergenerational transmission of adverse health consequences stemming from parental employment that can affect their children. With PE growing increasingly common in Sweden and elsewhere (11), it is important to advance the knowledge of the intergenerational effects that parental PE may have on children’s health. Such studies are especially urgent for adolescents where research is particularly scarce (12, 13), and for mental health, which is a common and increasing problem among children this age in Sweden (14).

In 2023, 12% of young men and 21% of young women aged 18–24 were either diagnosed with a mental health disorder or were prescribed psychotropic drugs in Sweden (15). Many mental health outcomes, such as anxiety and depression, are, to a large extent, socially determined (16) and therefore preventable. Thus, identifying key contributing factors can be helpful in efforts to improve the mental health of adolescents. Health in adolescence is often influenced by exposures in early childhood (17, 18), yet the significance of exposures later in childhood should not be neglected (18). While the school context and peers indeed have increasing influence on adolescents’ health and well-being, family factors continue to be of importance (19). Thus, for adolescent mental health, certain socially determined and avoidable exposures during this period may be particularly harmful. Especially, since experiencing socioeconomic disadvantage is known to affect the mental health of both children and adolescents (20), parental PE may be a contributing factor.

The current evidence on how parental PE influences the health of adolescents is scarce, although related research indicates that it may be a contributing factor to poorer health. For example, maternal non-standard working schedules have been associated with adolescent obesity (21) and maternal job insecurity and parental non-standard working schedules have been found to adversely influence adolescent mental health and depressive symptoms (12, 22). These findings are also in line with similar research focusing on younger children whose health and well-being can be linked to parental psychosocial working environment and employment quality (10, 23–26). However, these studies focus on working conditions and do not incorporate a multidimensional assessment of parental PE. The few studies containing such a multidimensional assessment of PE, show that maternal PE is associated with adolescent obesity (27), whereas parental PE has been shown to reduce adolescents’ happiness (28).

There are several plausible mechanisms, through which PE can negatively affect adolescent health. First, parental PE may influence poor health of adolescents through financial hardship, where material needs may be unmet and financial stress is increased (28). Secondly, parental psychosocial strain – such as feelings of vulnerability or fear of joblessness (28), stress (27) and poor parental mental health (29) – can also contribute to poor adolescent health. Moreover, stressful conditions at parents’ work and certain types of non-standard working schedules (eg, working late at night or during the weekends), can negatively influence family connectedness as both the time- and the quality of time spent with adolescents may be reduced (30, 31). With reduced family connectedness, parents are less able to monitor behaviors or to offer communication and support (31). This can increase the likelihood of developing adolescent problem behaviors (19, 30) such as sexual risk behaviors, substance misuse and truancy, which, in turn, are closely connected with adolescents’ mental health (32).

While evidence indicate that parental PE may affect the mental health of their adolescent children, the impact may differ between types of families. For example, two-parent families working non-standard working schedules may also find themselves having more time to spend with their children (12), which could be beneficial for adolescents’ mental health. Furthermore, the relationship between employment, children’s health, and family connectedness can be influenced by the parent’s gender, with outcomes for children varying depending on whether the exposure comes from the father or the mother (30, 31). These mixed relationships are likely due to an unequal burden of childcare responsibilities (33) and the different role that employment status has for the well-being of men and women (34). Ultimately, such gendered differences in burdens and social roles of the parents could influence the direction of the results for children’s health.

Despite this background, there is still limited research on how parental PE, measured as a multidimensional construct to fully grasp its complexity, associates with adolescents’ mental health over time and how this relationship is influenced by the gender of the parent. The aim of this study, therefore, was to assess the association between parental PE and the risk of their adolescent children developing a mental health disorder, with a particular focus on exploring the extent to which this relation is modified by the gender of the parent.

## Methods

### Study population

This register-based study uses data from the Swedish Work, Illness, and Labour-market Participation (SWIP) cohort. The SWIP cohort includes all individuals who were registered in Sweden and aged 16–65 in 2005. It is a closed cohort, meaning that no new participants are recruited at any later point in time. The SWIP cohort links information from multiple nationwide registries. It is anonymized and linked by Statistics Sweden. Here, sociodemographic and employment data comes from the Longitudinal Integration Database for Health Insurance and Labor Market Studies register (LISA); mental health disorder diagnoses, based on their ICD 10- and ICD 9-codes, were retrieved from the National Patient Registry; information on prescribed drugs comes from the Swedish Prescribed Drug Register; and linkage between children and parents is made possible through the multi-generation register.

The present study used a subpopulation of 117 437 adolescents followed from 2006–2009 (ie, until the year they turned 20 and exit adolescence), and at least one of their parents (adoptive or biological), whose sociodemographic information and employment conditions at baseline (2005) were assessed. Adolescents born in 1989, ie, aged 16 years at baseline, with at least one eligible parent, were selected. In turn, eligible parents were aged 35–67 years (ie, aged 19–51 the year when the child was born), and active in the labor market at baseline.

Exclusion criteria at baseline for the parents were: (i) missing information on the exposure (employment conditions), (ii) being a student, (iii) death, emigration or immigration, (iv) having a yearly employer-based income of <100 SEK (to ensure labor market attachment), (v) being self-employed, to avoid misclassification to PE, (vi) >90 days of unemployment, to avoid measuring the effect of unemployment on the outcome, (vii) being on long-term sick leave (>180 days), (viii) being early retired or (ix) being a pensioner. At least one parent had to fit the eligibility criteria.

Exclusion criteria for the adolescents at baseline were: (i) any healthcare visit prior to 2006 due to mental health disorder, based on diagnosis (ICD 10: F00–F99; ICD 9: 291–319), including self-harm or suicide attempts (ICD 10: X60-X84; ICD 9: E95, E98), and/or (ii) prescriptions of psychotropic drugs in 2005 (ATC-codes: N06A, N05B and N05C).

Since eligibility was required for only one parent, a strategy was needed to ensure that adolescents with an ineligible second parent were still included in the analyses. For this reason, additional categories were created, consisting of two groups: others and not available (NA). The latter comprised individuals who either did not have a second parent or whose second parent was not recorded in the LISA registry. The ‘other’ group consisted of parents who did not meet the inclusion criteria.

### Exposure

Parental PE was estimated based on the Swedish Register-based Operationalization of Precarious Employment 2.0 (SWE-ROPE) (35, 36). SWE-ROPE uses five items (contractual employment insecurity, contractual temporariness, multiple-job holding, income level and collective bargaining agreement coverage) (35) to capture the three dimensions of PE (36): employment insecurity, income inadequacy, and a lack of rights and protections (9) as shown in table 1. Item scores are summed, and the employment score of individuals can range from -9 to +2. PE is identified by scores < -3; standard employment (SE) is identified by scores ≥0; and the middle group consisting of scores of -3– -1 represents those with substandard employment (SSE). SE was used as a reference group, to which parents in PE and SSE were compared.

**Table 1:** Swedish register-based operationalization of precarious employment 2.0 (SWE-ROPE) scoring of items.

Item	Score				
	-2	-1	0	1	2
Contractual employment insecurity		Agency employed	Directly employed		
Temporariness	Unstable employment		Stable employment		
Multiple-job holding	Multiple jobs (>2) and sectors (>1)	Multiple jobs (>2 jobs)	No multiple jobs (1 job)		
Income level (% of median)	<60	60–80	81–120	121–200	>200
Covered by collective bargaining agreement (% likelihood)	<70	70–90	91–100		

### Outcome

The outcome of interest was adolescents’ mental health disorders assessed as the first-time admission during follow-up with a mental disorder in either inpatient or specialized outpatient registries. The outcome contained the following disorders (ICD 10 codes in brackets): depression (F32–F33), anxiety (F41), stress-related disorders (F43), suicide attempt – intentional self-harm (X60-X84) and eating disorders (F50). The rationale for the selection of these diagnoses was to focus on both common mental health disorders (such as depression and anxiety disorders) as well as disorders with a high prevalence among adolescents that were highly interconnected to common mental disorders (such as self-harm or eating disorders). As an additional strategy to identify mental health disorders among adolescents who may not have been diagnosed in either inpatient or specialized outpatient care, the first incidence of treatment during follow-up with psychotropic drugs was also assessed. These were identified by their ATC codes from the prescribed drug register and included antidepressants (N06A), anxiolytics (N05B) and hypnotics and sedatives (N05C). The outcome was assessed dichotomously as either having a mental health disorder (the incidence of diagnosis or prescribed drug) or not (37).

### Covariates

Covariates were measured in 2005. For the adolescents, these included family type (living in a household with two parents, single father or single mother), number of siblings, and parental and individual migrant background (born in Sweden to two Swedish parents, born in Sweden to one foreign parent, born in Sweden to two foreign parents, and foreign-born). Parental covariates included parental employment, age and highest educational attainment among the parents (in the case of two parents). Parental history of a mental health diagnosis was also assessed in sensitivity analyses, with practically identical results. Given that it could be a mediator in the association between parental PE and adolescents’ mental health, it was not included it in the main analyses.

In this study, adolescents’ own potential employment was not included as a covariate for a variety of reasons. Firstly, any potential job would be at the side of studying as close to all 16-year-olds in Sweden attended high school in 2005 (37). Furthermore, it is not possible to distinguish between those who work to earn some extra pocket money and those who work out of necessity, but both cases of adolescent employment are likely dependent on parental education and employment and should thus be understood as a mediator rather than a confounder. Finally, the operationalization of PE used in this study partly relies on assessing employment stability over two years, meaning that only those working two years prior to the age of 16 would be included, which makes it unfeasible given the data availability.

### Data analysis

First, baseline characteristics of the adolescents and their parents based on the selected covariates, were assessed and presented. Then, Cox regression models were applied to estimate the hazard ratios (HR) with 95% confidence intervals (CI), for the outcome as dependent on parental employment as either PE, SSE or SE. Person-time is calculated from 1 January 2006 until the first incidence of the outcome of mental health disorders, until the year they turn 20 (2009), emigration or death, whichever one comes first. Model 1 was adjusted for the employment of the other parent (if there was more than one), and Model 2 was additionally adjusted for migrant status, number of siblings, family type, parents’ age in 2005 and highest parental educational attainment. Sensitivity analyses were conducted to test the robustness of the results. Here the results were stratified by the gender of the parent, stratified by the gender of the child, and parental history of mental health disorders was added as a covariate. All analyses have been conducted using STATA version 18, Stata Corp, College Station, TX, USA.

## Results

### Baseline characteristics

Table 2 shows the baseline characteristics of the adolescents and their parents. Most adolescents were born in Sweden to Swedish-born parents (76.86%) and lived in two-parent households (70.31%). Most of the parents were aged 40–49 at baseline (63.71% by mothers and 55.89% by fathers), meaning they were between the ages of 24–33 the year the child was born. The highest educational attainment of the parents was most frequently reported as middle educational attainment (48.98%). In terms of parental employment, SE was the most common employment type among both mothers (39.60%) and fathers (47.84%). PE constituted the smallest group (3.75% among mothers and 2.89% among fathers). The sample of parents in PE in this study is lower than for the general population (11), which was expected since precariousness often leads to a postponement of family formation (38).

**Table 2:** Characteristics of the study sample at the 2005 baseline (N=117 437). [NA=not available; SE=standard employment; SSE=substand employment; PE=precarious employment.]

			N	%
Characteristics of adolescents				
	Sex			
		Male	60 537	51.55
		Female	56 900	48.45
	Migrant background			
		Foreign born	9027	7.69
		Born in Sweden, two foreign parents	6977	5.94
		Born in Sweden, one foreign parent	11 175	9.52
		Born in Sweden, Swedish parents	90 258	76.86
	Family type			
		Lives with two parents	82 566	70.31
		Lives with single mother	27 735	23.62
		Lives with single father	7136	6.08
	Number of siblings ^a^			
		0	20 895	17.79
		1	53 619	45.66
		2	30 265	25.77
		≥3	12 628	10.75
	Characteristics of parents			
	Mothers’ age (years)			
		35–39	19 383	16.51
		40–49	74 824	63.71
		50–59	18 914	16.11
		60–67	238	0.20
		NA ^c^	4078	3.47
	Fathers’ age			
		35–39	7760	6.61
		40–49	65 639	55.89
		50–59	30 961	26.36
		60–67	2940	2.50
		NA ^c^	10 137	8.63
	Highest parental education ^b^			
		Low	8085	6.88
		Middle	57 524	48.98
		High	51 346	43.72
	Mothers’ employment			
		SE	46 509	39.60
		SSE	28 231	24.04
		PE	4399	3.75
		Other ^c^	34 213	29.13
		NA ^c^	4085	3.48
	Fathers’ employment			
		SE	56 185	47.84
		SSE	13 430	11.44
		PE	3396	2.89
		Other ^c^	34 284	29.19
		NA ^c^	10 142	8.64
	Mothers’ history of mental health disorders			
		No	109 320	93.09
		Yes	4032	3.43
		NA ^c^	4085	3.48
	Fathers’ history of mental health disorders			
		No	104 466	88.95
		Yes	2829	2.41
		NA ^c^	10 142	8.64

^a^ Missing: 30 (0.03%) missing in number of siblings. ^b^ 482 (0.41%) missing in highest parental education. ^c^ The categories other and NA refer to ineligible second parents who had to be included for methodological reasons. See the methods section for a more detailed explanation.

### Associations between parental PE and adolescent mental health disorders

During the follow-up period, there were 13 095 incident cases of mental health disorders, affecting 11.2% of our sample of adolescents. Mental disorders were significantly more common among adolescents with parents in PE (father: 12.7%, mother: 11.4%) compared to adolescents with parents in SE (father: 10.1%, mother: 10.0%) (table 3).

**Table 3:** Crude and adjusted hazard ratios (HR) with 95% confidence intervals (CI) for the association between parental precarious employment (PE, 2005) and adolescent mental health disorders (2006–2009). [SE=standard employment; SSE=substand employment.]

		Exposed (N)/Case (%)	Model 1 ^a^			Model 2 ^b^	
			HR	95% CI		HR	95% CI
Mothers’ employment							
	SE (ref)	46 509/10.02	1.00			1.00	
	SSE	28 231/10.18	1.01	0.96–1.05		1.01	0.96–1.06
	PE	4399/11.39	1.11 *	1.00–1.21		1.12 *	1.02–1.22
Fathers’ employment							
	SE (ref)	56 185/10.07	1.00			1.00	
	SSE	13 430/11.14	1.09 **	1.03–1.15		1.06 *	1.00–1.12
	PE	3396/12.72	1.22 **	1.11–1.35		1.17 **	1.06–1.29

^a^ Model 1 adjusts for the employment of the other parent. ^b^ Model 2 adjusts for the employment of the other parent, migrant background, number of siblings, mothers- and fathers’ age in 2005, highest parental education and family type. ** P<0.01. * P<0.05.

Table 3 shows the association between parental PE and adolescent mental health disorders as compared to adolescents with parents in SE. Model 1 shows that adolescents with either a mother (HR 1.11, 95% CI 1.00–1.21) or a father in PE (HR 1.22, 95% CI 1.11–1.35) had a statistically significantly increased risk of developing mental health disorders. After adjusting for all covariates, the risk among adolescents to develop mental health disorders slightly increased as associated with maternal PE (HR 1.12, 95% CI 1.02–1.22) and reduced slightly for the association with paternal PE (HR 1.17, 95% CI 1.06–1.29). Overall, the results indicated that especially fathers’ PE was associated with an increased risk of developing mental health disorders for adolescents.

As seen in model 2, while adolescents to fathers in SSE also had a slight increased risk of developing mental health disorders compared to those with fathers in SE (HR 1.06, 95% CI 1.00–1.12), this association was not found for those with mothers in SSE (HR 1.01, 95% CI 0.96–1.06). Finally, it should be noted that the CI were close to one and that the differences between mothers’ and fathers in PE were small.

### Sensitivity analyses

Several sensitivity analyses were conducted to test the robustness of the results. First, the results were stratified by the gender of the parent (supplementary material, www.sjweh.fi/article/4210, table S1). The results for the association between parental PE and adolescents’ mental health remained similar although slightly attenuated for both mothers (HR 1.13, 95% CI 1.03–1.24) and fathers (HR 1.20, 95% CI 1.09–1.32). Then, parental history of mental health disorders was added as a covariate (supplementary table S2). Again, the overall results for PE remained the same although this time slightly reduced (mothers: HR 1.11, 96% CI 1.01–1.22; fathers: HR 1.16, 95% CI 1.05–1.28). Finally, the results were stratified by the gender of the adolescent. Here, the effect of parental PE was somewhat stronger for boys than for girls and the only significant result was for fathers PE increasing the risk for boys to develop mental health disorders (supplementary table S3). However, after investigating this further, a model with interaction terms (model not shown) between parental PE and the gender of the child, showed that these differences were not statistically significant. The stratified results may thus be due to a loss of power as the sample was significantly reduced.

## Discussion

To our knowledge, this is the first longitudinal study using high-quality registry data to investigate how parental PE, measured as a multidimensional construct, associates with the risk of mental health disorders of their children in late adolescence. The results suggest that being an adolescent to a parent in PE slightly increases the risk of developing mental health disorders, primarily among adolescents whose fathers are precariously employed. Overall, these results remained robust after having conducted several sensitivity analyses. Thus, this study is an important contribution to the evidence concerning the intergenerational transmission of disadvantages in mental health stemming from PE.

The first objective of this study was to assess how parental PE is associated with the mental health of adolescents, and the main results showed that parental PE associates with an increased risk for adolescents to develop mental health disorders. This finding is in line with similar studies that, although often based on smaller sample sizes, have also found that parental PE and associated exposures, such as non-standard working schedules, can impact the health and well-being of adolescents negatively (12, 21, 22, 27, 28). The results could be attributed to increased parental financial strain, work–family conflict, reduced family connectedness, parental psychosocial strain, or parental mental health disorders that could spillover and harm the health and well-being of children (28, 31). While future research is needed to test these pathways, the study results support the argument that family factors are of continued importance also for adolescents and not only younger children (19), here in terms of mental health and parental employment quality.

The second objective of this study was to explore how this association varies if the father or mother is in PE. While differences were small, the results suggested that adolescents with fathers in PE had the highest risk of developing mental health disorders. The current evidence is mixed regarding how the health and well-being of children differ based on the employment quality of mothers and fathers. There is more evidence for harmful effects of maternal employment due to a lack of studies specifically on fathers’ employment (22, 24, 27, 39). Among studies that consider employment and work for both parents, mother’s exposure to low job quality (10) and father’s work-related factors (23, 26) have each been found to be especially associated with the health and well-being of children. It is therefore crucial to apply a contextual understanding of the gendered impact of employment on children’s health, and potential explanations for the findings of this study are discussed below from a gendered perspective.

The finding that the risk for developing mental health disorders among adolescents was somewhat higher for those with fathers’ in PE, could be explained by social role theory, which suggests that various employment situations are experienced differently by men and women due to the interplay between normative gender roles and employment. For example, men’s well-being (such as life satisfaction or happiness) is reduced to a greater extent in the case of unemployment than it is for women (34) allegedly due to the unmet normative expectation of the male family breadwinner. It has been suggested that parental well-being influences their relationship with their adolescent children and the family connectedness. And with reduced family connectedness, adolescent risk behaviors can develop (30) which can lead to adverse mental health, making it a plausible explanation for our results. Women’s well-being, on the other hand, may not suffer equally from unemployment or PE as their social value is more commonly ascribed to them through their role as a mother or wife (40), a role they may perform even better when not working standard full-time jobs.

However, it is also likely that compared to men in PE, women in PE make greater efforts to find time to fulfil their role as caregivers and contribute to greater family connectedness, despite facing challenges due to their employment (23) and regardless of how this affects their own well-being. Thus, the fact that adolescents with fathers in PE have a higher risk of developing mental health disorders may suggest that, in Sweden, the traditional role of men as the family breadwinner persists and that fathers in PE then may suffer psychosocially when this role is unfulfilled, which can spillover and influence adolescents. For mothers, however, social role theory may not explain the findings as well, since compared to adolescents with mothers in SE, adolescents with mothers in PE still had an increased risk of mental health disorders.

In turn, a strictly financial pathway can also offer support to the results of this study. Women in Sweden generally earn less compared to men (41). Thus, fathers in PE could have a stronger influence on the mental health of adolescents because having a father in PE, compared to a father in SE, could mean a greater absolute financial reduction for the household compared to having a mother in PE versus SE. If women generally earn less, the absolute wage gap between women in various employment categories may not differ as much as it may do for men, which could also explain why we found only paternal SSE and not maternal SSE, to increase the risk of adolescents developing mental health disorders. While the financial pathway of a parent in PE can influence adolescent mental health, this influence is not necessarily determined solely by the parent’s gender. The overall household income is also likely a crucial factor in shaping adolescent mental health.

Finally, the results of this study remained robust even after conducting several sensitivity analyses. For example, in this study, parental mental health diagnoses were examined as a potential confounding factor between parental PE and adolescent mental health, but it did not affect the main results. Regarding the gender of the adolescent, it initially seemed that the mental health of boys with fathers in PE were at greater risk compared to girls with parents in PE. However, after further investigations, these results were not significant. While these results are too inconclusive to add any evidence to this topic, it does, as has been suggested by others (42), potentially implicate that the gendered interaction of both parents and children may be of relevance for the link between parental PE and children’s health. This should be investigated in more detail in future research on this topic.

### Strengths and limitations

A strength of the study is the use of high-quality register-based data. Firstly, this allows for more objective measures of both the exposure and the outcome, which are otherwise often self-reported. Secondly, this register-based study allows for a relatively large sample size and a longitudinal approach, both of which are scarce in existing research on this topic. Moreover, similar studies often focus exclusively on children living in either sole-parent households (12), two-parent households (10, 43) or they focus on the exposure measured only by the mother (21, 27, 29). This current study, however, includes children with one or two parents and considers both paternal and maternal employment, which allows an investigation of a greater diversity in family formations. Finally, SWE-ROPE allows for considering PE as a multidimensional construct rather than looking only at a single dimension.

Some limitations of this study stem from data restrictions. First, the SWIP cohort includes only those aged ≥16 in 2005. While adolescence traditionally includes the ages 10–19 (44), the data used here restricted the scope of children to those in their later adolescence. Still, late adolescence is currently the most overlooked age group in similar research, illustrating the relevance to focus on this group. Furthermore, the fact that no new recruitments are made to the SWIP cohort, means that 2005 was the most recent year that these 16-year-old children could be included. In Sweden around 2005, both PE (11) and adolescent mental health diagnoses (45) were rarer than they are today. As the current study found a relationship between the two, an even stronger association could be expected when using more recent data, highlighting the importance of these findings. Second, history of mental health diagnoses prior to follow-up used in sensitivity analysis (in the case of parents) and as exclusion criteria (in the case of adolescents) was assessed strictly through inpatient data. Due to register constraints, we were unable to include information from the specialized outpatient care register, which reached complete coverage in 2003, or prescribed drug registry, which started in 2005. Last, due to data limitations of the SWIP cohort, our focus on adolescents meant that PE could only be measured at baseline (2005), preventing an assessment of employment trajectories. Further research should incorporate a longitudinal assessment of PE as it may influence the relationship.

### Concluding remarks

This study found an association between parental PE and the mental health of their adolescent children, and primarily among adolescents whose fathers are precariously employed. These findings support the assumption that there is an intergenerational transmission of disadvantage linked to lower employment quality among parents. As PE becomes increasingly common, this study emphasizes the urgent need for policies and regulations addressing PE and its harmful impact given its detrimental effects not only on the health of workers but also on their adolescent children’s mental well-being.

## Supplementary material

Supplementary material

## References

[1] Benach J, Vives A, Amable M, Vanroelen C, Tarafa G, Muntaner C. Precarious employment: understanding an emerging social determinant of health. Annu Rev Public Health 2014;35:229–53. 10.1146/annurev-publhealth-032013-182500 24641559

[2] Matilla-Santander N, Muntaner C, Kreshpaj B, Gunn V, Jonsson J, Kokkinen L et al. Trajectories of precarious employment and the risk of myocardial infarction and stroke among middle-aged workers in Sweden: A register-based cohort study. Lancet Reg Health Eur 2022 Feb;15:100314. 10.1016/j.lanepe.2022.100314 35169764 PMC8829810

[3] Julià M, Méndez-Rivero F, Gómez-Gómez Á, Pozo ÓJ, Bolíbar M. Association between precarious employment and chronic stress: effect of gender, stress measurement and precariousness dimensions—a cross-sectional study. Int J Environ Res Public Health 2022 Jul;19(15):9099. 10.3390/ijerph19159099 35897463 PMC9330896

[4] Pförtner TK. The emergence of precarious employment as a determinant of health in europe and the relevance of contextual factors: a critical research synthesis. Int J Soc Determinants Health Health Serv 2023 Jul;53(3):266–81. 10.1177/00207314221139797 36444760

[5] Pollack R, Kreshpaj B, Jonsson J, Bodin T, Gunn V, Orellana C et al. Low-quality employment trajectories and the risk of common mental health disorders among individuals with Swedish and foreign background - a register-based cohort study. Scand J Work Environ Health 2022 Jul;48(5):351–60. 10.5271/sjweh.4029 35546057 PMC9527776

[6] Rönnblad T, Grönholm E, Jonsson J, Koranyi I, Orellana C, Kreshpaj B et al. Precarious employment and mental health: a systematic review and meta-analysis of longitudinal studies. Scand J Work Environ Health 2019 Sep;45(5):429–43. 10.5271/sjweh.3797 31165899

[7] Rugulies R, Bültmann U, Aust B, Burr H. Psychosocial work environment and incidence of severe depressive symptoms: prospective findings from a 5-year follow-up of the Danish work environment cohort study. Am J Epidemiol 2006 May;163(10):877–87. 10.1093/aje/kwj119 16571741

[8] Jonsson J, Muntaner C, Bodin T, Alderling M, Rebeka R, Burström B et al. Low-quality employment trajectories and risk of common mental disorders, substance use disorders and suicide attempt: a longitudinal study of the Swedish workforce. Scand J Work Environ Health 2021 Oct;47(7):509–20. 10.5271/sjweh.3978 34397098 PMC8504160

[9] Kreshpaj B, Orellana C, Burström B, Davis L, Hemmingsson T, Johansson G et al. What is precarious employment? A systematic review of definitions and operationalizations from quantitative and qualitative studies. Scand J Work Environ Health 2020 May;46(3):235–47. 10.5271/sjweh.3875 31901944

[10] Nicholson JM, Strazdins L, Brown JE, Bittman M. How parents’ income, time and job quality affect children’s health and development. Aust J Soc Issues 2012;47(4):505–25. 10.1002/j.1839-4655.2012.tb00263.x

[11] Bodin T, Matilla-Santander N, Selander J, Gustavsson P, Hemmingsson T, Johansson G et al. Trends in precarious employment in Sweden 1992–2017: a social determinant of health. Int J Environ Res Public Health 2022 Oct;19(19):12797. 10.3390/ijerph191912797 36232108 PMC9565988

[12] Dockery A, Li J, Kendall G. Parents’ work patterns and adolescent mental health. Soc Sci Med 2009 Feb;68(4):689–98. 10.1016/j.socscimed.2008.10.005 19084312

[13] Arlinghaus A, Bohle P, Iskra-Golec I, Jansen N, Jay S, Rotenberg L. Working Time Society consensus statements: evidence-based effects of shift work and non-standard working hours on workers, family and community. Ind Health 2019 Apr;57(2):184–200. 10.2486/indhealth.SW-4 30700670 PMC6449634

[14] Public health agency of Sweden. Varför har den psykiska ohälsan ökat bland barn och unga i Sverige? Utvecklingen under perioden 1985− 2014. [Why Has Mental Illness Increased among Children and Young People in Sweden? Developments during the Period 1985–2014] Folkhälsomyndigheten; Stockholm, Sweden: The Public Health Agency of Sweden; 2018.

[15] the National Board of Health and Welfare. Förekomst av psykisk ohälsa bland barn och unga vuxna: Aspekter av socioekonomiska utmaningar och förutsättningar. [Occurence of mental illness among children and young adults: Aspects of socioeconomic challenges and prerequisits]. Socialstyrelsen 2024.

[16] Alegría M, NeMoyer A, Falgàs Bagué I, Wang Y, Alvarez K. NeMoyer A, Falgàs Bagué I, Wang Y, Alvarez K. Social determinants of mental health: where we are and where we need to go. Curr Psychiatry Rep 2018 Sep;20(11):95. 10.1007/s11920-018-0969-9 30221308 PMC6181118

[17] Hynek KA, Abebe DS, Hollander AC, Liefbroer AC, Hauge LJ, Straiton ML. The association between persistent low parental income during preschool age and mental disorder in adolescence and early adulthood: a Norwegian register-based study of migrants and non-migrants. BMC Psychiatry 2022 Mar;22(1):206. 10.1186/s12888-022-03859-6 35305586 PMC8934484

[18] Sawyer SM, Afifi RA, Bearinger LH, Blakemore SJ, Dick B, Ezeh AC et al. Adolescence: a foundation for future health. Lancet 2012 Apr;379(9826):1630–40. 10.1016/S0140-6736(12)60072-5 22538178

[19] Viner RM, Ozer EM, Denny S, Marmot M, Resnick M, Fatusi A et al. Adolescence and the social determinants of health. Lancet 2012 Apr;379(9826):1641–52. 10.1016/S0140-6736(12)60149-4 22538179

[20] Reiss F. Socioeconomic inequalities and mental health problems in children and adolescents: a systematic review. Soc Sci Med 2013 Aug;90:24–31. 10.1016/j.socscimed.2013.04.026 23746605

[21] Kachi Y, Abe A, Eguchi H, Inoue A, Tsutsumi A. Mothers’ nonstandard work schedules and adolescent obesity: a population-based cross-sectional study in the Tokyo metropolitan area. BMC Public Health 2021 Jan;21(1):237. 10.1186/s12889-021-10279-w 33509145 PMC7845102

[22] Mauno S, Cheng T, Lim V. The far-reaching consequences of job insecurity: A review on family-related outcomes. Marriage Fam Rev 2017;53(8):717–43. 10.1080/01494929.2017.1283382

[23] Kim M. Parental nonstandard work schedules and child development: evidence from dual-earner families in Hong Kong. Int J Environ Res Public Health 2021 May;18(10):5167. 10.3390/ijerph18105167 34068105 PMC8152747

[24] Schneider D, Harknett K. Maternal exposure to work schedule unpredictability and child behavior. J Marriage Fam 2022 Feb;84(1):187–209. 10.1111/jomf.12800 35874104 PMC9293031

[25] Walther AK, Pilarz AR. Associations between parental precarious work schedules and child behavior problems among low‐income families. J Marriage Fam 2023.

[26] Zilanawala A, Abell J, Bell S, Webb E, Lacey R. Parental nonstandard work schedules during infancy and children’s BMI trajectories. Demogr Res 2017;37:709–26. 10.4054/DemRes.2017.37.22

[27] Zhuang CC, Jones-Smith JC, Andrea SB, Hajat A, Oddo VM. Maternal precarious employment and child overweight/obesity in the United States. Prev Med 2023 Apr;169:107471. 10.1016/j.ypmed.2023.107471 36870570 PMC10041450

[28] Han WJ, Hart J. Precarious parental employment, economic hardship, and parenting and child happiness amidst a pandemic. Child Youth Serv Rev 2022;133:106343. 10.1016/j.childyouth.2021.106343

[29] Mauno S, Minkkinen J, Hirvonen R, Kiuru N. Job insecurity and depressive symptoms in mothers and adolescents: a dyadic study. J Child Fam Stud 2021;30(9):2117–28. 10.1007/s10826-021-01994-4

[30] Han WJ, Miller DP, Waldfogel J. Parental work schedules and adolescent risky behaviors. Dev Psychol 2010 Sep;46(5):1245–67. 10.1037/a0020178 20822236 PMC3742548

[31] Täht K, Mills M. Out of time: The consequences of non-standard employment schedules for family cohesion: Springer; 2015.

[32] Kaess M, Brunner R, Parzer P, Carli V, Apter A, Balazs JA et al. Risk-behaviour screening for identifying adolescents with mental health problems in Europe. Eur Child Adolesc Psychiatry 2014 Jul;23(7):611–20. 10.1007/s00787-013-0490-y 24248753

[33] Wondemu MY, Joranger P, Hermansen Å, Brekke I. Impact of child disability on parental employment and labour income: a quasi-experimental study of parents of children with disabilities in Norway. BMC Public Health 2022 Sep;22(1):1813. 10.1186/s12889-022-14195-5 36151541 PMC9508753

[34] Van der Meer PH. Gender, unemployment and subjective well-being: why being unemployed is worse for men than for women. Soc Indic Res 2014;115(1):23–44. 10.1007/s11205-012-0207-5

[35] Bodin T, Jonsson J. Swedish Register Operationalization of Precarious Employment (SWE-ROPE) GitHub. 2020 Available from: https://github.com/TBodin/swe-rope

[36] Jonsson J, Matilla-Santander N, Kreshpaj B, Orellana C, Johansson G, Burström B et al. Exploring multidimensional operationalizations of precarious employment in Swedish register data - a typological approach and a summative score approach. Scand J Work Environ Health 2021 Mar;47(2):117–26. 10.5271/sjweh.3928 32997147 PMC8114571

[37] Skolverket. Beskrivande data om förskoleverksamhet, skolbarnsomsorg, skola och vuxenutbildning, 2007 [Descriptive data on preschool activities, school-age childcare, schools and adult education in Sweden, 2007]. Stockholm: Skolverket/The National Agency for Education; 2007.

[38] van Wijk DC, de Valk HA, Liefbroer AC. Economic precariousness and the transition to parenthood: A dynamic and multidimensional approach. Eur J Popul 2022 Apr;38(3):457–83. 10.1007/s10680-022-09617-4 35966358 PMC9363546

[39] Patil D, Enquobahrie DA, Peckham T, Seixas N, Hajat A. Retrospective cohort study of the association between maternal employment precarity and infant low birth weight in women in the USA. BMJ Open 2020 Jan;10(1):e029584. 10.1136/bmjopen-2019-029584 31924630 PMC6955480

[40] Tattarini G, Grotti R. Gender roles and selection mechanisms across contexts: a comparative analysis of the relationship between unemployment, self-perceived health and gender. Sociol Health Illn 2022 Mar;44(3):641–62. 10.1111/1467-9566.13449 35218011

[41] Boye K, Halldén K, Magnusson C. Stagnation only on the surface? The implications of skill and family responsibilities for the gender wage gap in Sweden, 1974-2010. Br J Sociol 2017 Dec;68(4):595–619. 10.1111/1468-4446.12252 28369726

[42] Li M, Chzhen Y. Insecure maternal employment and children’s behaviour difficulties: evidence from the longitudinal study of Australian children. Soc Sci Med 2024 Aug;354:117077. 10.1016/j.socscimed.2024.117077 38976938

[43] Prickett KC. Nonstandard work schedules, family dynamics, and mother–child interactions during early childhood. J Fam Issues 2018 Mar;39(4):985–1007. 10.1177/0192513X16684893 29651191 PMC5891148

[44] World Health Organization. Guidelines on mental health promotive and preventive interventions for adolescents: helping adolescents thrive: World Health Organization; 2020. 33301276

[45] Dalman CB, S; Åhlén, J; Ohlis, A; Agardh, E; Wicks, S; Lundin, A. Mental well-being, mental distress, and mental disorders among children and young adults - terminology, measurement methods, and prevalence, an overview. Forte - forskningsrådet för hälsa, arbetsliv och välfärd; 2021.

