# Supplementary material for: Parental precarious employment and the mental health of adolescents: a Swedish registry study
Source: Scand J Work Environ Health. 2025 Feb 28;51(2):59–67. doi: 10.5271/sjweh.4210 (PMC11886878; doi:10.5271/sjweh.4210)
Supplement: Supplementary material [file SJWEH-51-59-S001.pdf]

Parental precarious employment and the mental health of adolescents: a Swedish registry study[1]

by Amanda E Aronsson, MSc[2] Emelie Thern, PhD, Nuria Matilla-Santander, PhD, Signild Kvart, MSc, Julio C Hernando-Rodriguez, PhD, Kathryn Badarin, PhD, Mireia Julià, PhD, Samira Alfayumi-Zeadna, PhD, Virginia Gunn, PhD, Bertina Kreshpaj, PhD, Carles Muntaner, PhD, Theo Bodin, PhD, Lluís Mangot-Sala, PhD

1. Supplementary material
2. Correspondence to: Amanda E. Aronsson, Department of Sociology and Political Science, Norwegian University of Science and Technology, postbox 8900, Torgarden, 7491 Trondheim, Norway. [E-mail: Amanda.aronsson@ntnu.no]

Figure 1. Flow chart of analytical sample.

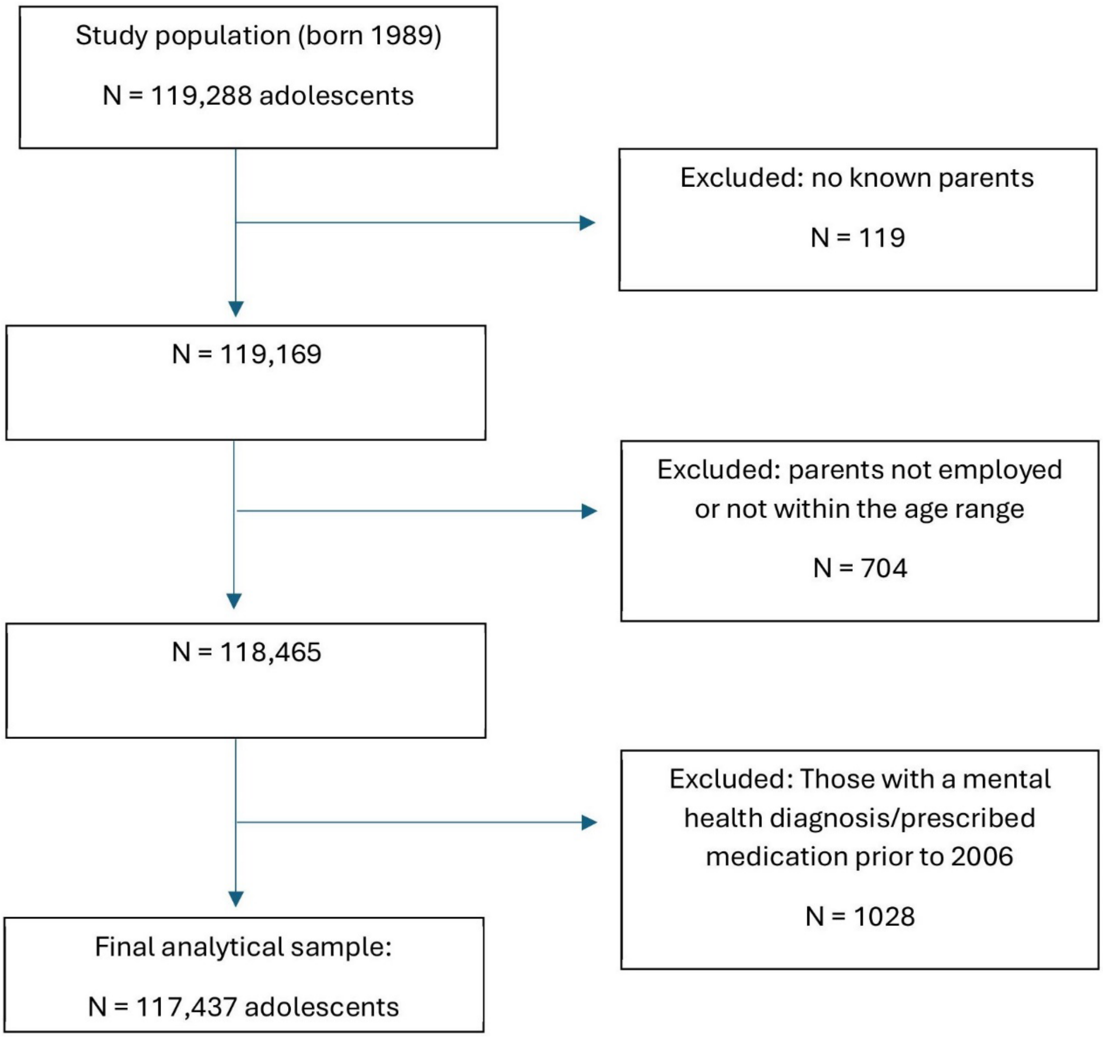

Supplementary Table S1. Adjusted hazard ratios (HRs) with 95% confidence intervals (CIs) for the association between parental PE (2005) and adolescent mental health disorders (2006-2009). Stratified by mothers and fathers

	Mothers[a]		Fathers[b]	
	HR	95% CI	HR	95% CI
**SE (ref)**	1.00		1.00	
**SSE**	1.02	0.97-1.07	1.08*	1.01-1.14
**PE**	1.13**	1.03-1.24	1.20**	1.09-1.32

[a]Adjusted for migrant background, number of siblings, mothers age in 2005, highest maternal education and family type

[b]Adjusted for migrant background, number of siblings, fathers age in 2005, highest paternal education and family type

** $p < 0.01$, *$p < 0.05$

Supplementary Table S2. Adjusted hazard ratios (HRs) with 95% confidence intervals (CIs) for the interaction effect between parental PE (2005) and adolescent mental health disorders (2006-2009). Additionally adjusted for by the history of parental severe mental health issues.

	HR	95% CI
**Mothers' employment**		
SE (ref)	1.00	
SSE	1.01	0.96-1.06
PE	1.11*	1.01-1.22
**Fathers' employment**		
SE (ref)	1.00	
SSE	1.06	1.00-1.12
PE	1.16**	1.05-1.28

Adjusted for the employment of the other parent, migrant background, number of siblings, mothers- and fathers' age in 2005, highest parental education, family type and parental history of severe mental health issues

** $p < 0.01$, *$p < 0.05$

Supplementary Table S3. Adjusted hazard ratios (HRs) with 95% confidence intervals (CIs) for the association between parental PE (2005) and adolescent mental health disorders (2006-2009). Stratified by the gender of the child

	Exposed (n)/ Case (%)	**Boys**		Exposed (n)/ Case (%)	**Girls**	
		**HR**	**95% CI**		**HR**	**95% CI**
**Mothers' employment**						
SE (ref)	23 904/7.04	1.00		11 605/13.18	1.00	
SSE	14 546/7.48	1.06	0.98-1.15	13 685/13.05	0.98	0.92-1.04
PE	2289/8.39	1.15	0.99-1.34	2110/14.64	1.10	0.97-1.24

**Fathers' employment**						
SE (ref)	29 006/7.03	1.00		27 179/13.32	1.00	
SSE	6904/7.85	1.07	0.97-1.18	6526/14.62	1.05	0.98-1.13
PE	1728/9.61	1.24**	1.06-1.45	1668/15.95	1.12	0.99-1.27

Adjusted for the employment of the other parent, migrant background, number of siblings, mothers- and fathers' age in 2005, highest parental education and family type

** $p < 0.01$, *$p < 0.05$
